# The native Iranian soil bacteria with high potential to produce extracellular methionine gamma-lyase

**DOI:** 10.3389/fmicb.2024.1504742

**Published:** 2024-12-12

**Authors:** Matin Nasirian, Mohsen Mobini-Dehkordi, Pegah Khosravian

**Affiliations:** ^1^Genetics Department, Faculty of Basic Science, Shahrekord University, Shahrekord, Iran; ^2^Medical Plants Research Center, Basic Health Sciences Institute, Shahrekord University of Medical Sciences, Shahrekord, Iran

**Keywords:** L-Methioninase, cancer, native bacteria, optimization process, fermentation

## Abstract

This study aimed to screen native methionine gamma-lyase (L-methioninase) producing bacteria from soil samples and optimize the culture media for enhanced enzyme production using statistical design. Three bacteria, *Pseudomonas mosselii, Ralstonia solanacearum, and Cytobacillus kochii,* were identified as novel L-methioninase producers, which alternative source of L-methioninase for cancer treatment could be utilized alongside other therapeutic agents. The bacteria were isolated from various garden soils and cultured on a modified M9 medium and screened by Nessler reagent. According to Bergey’s manual of systematic bacteriology, identification tests determined the morphological, physiological, and biochemical characterizations. Further identification was performed using the analysis of the 16 s rDNA gene sequences using PCR and universal bacterial primers. The optimization of medium constituents for L-methioninase production was performed in two steps using Response Surface Methodology (RSM). The first step used the “one factor at a time” method to screen and identify critical medium components for L-methioninase production. The second step used the Box–Behnken design to assess quadratic effects and two-way interactions between variables and determine the response’s nonlinear nature. The study found that three isolates produced L-methioninase, namely *Pseudomonas mosselii* spp.*MN02* (GenBank PP431975), *Ralstonia solanacearum* spp.*MN02* (GenBank PP431636), and *Cytobacillus kochii* spp.*MN02* (GenBank PP432622). Among these, *Pseudomonas mosselii* spp.*MN02* produced the highest amount of L-methioninase and was therefore chosen for enzyme production optimization process. The maximum L-methioninase production of 1.5 ± 0.1 U/mL was obtained at a pH 6, and the best nitrogen source was yeast extract (1% concentration). The influence of different carbon sources revealed that glucose was the best carbon source for L-methioninase production (3.25 ± 0.1 U/mL). The optimization experiments using the Box–Behnken design predicted that L-methioninase would have an activity of 12.56 U/mL under optimal conditions, including 2% glucose, 2% yeast extract, pH 6, and temperature at 30°C. In conclusion, this study presents a promising new methods for identifying potential L-methioninase producers and optimizing the culture medium for more enzyme production by microbial fermentation. This could pave the way for developing a drug that assists in human cancers treatment.

## 1 Introduction

Methionine gamma-lyase or L-methioninase is an enzyme dependent on pyridoxal 5′-phosphate as cofactor. L-methioninase is an enzyme that depletes methionine from the blood, so cancer cells move toward cell death due to a lack of methionine. It is a Hoffman effect (Abozeid, 2023). This enzyme’s mechanism of action is that methionine’s anti-tumor mechanism is due to the loss of the function of molecules synthesized from methionine. According to research and newly published articles, methioninase is effective on all types of cancer (Siblini et al., 2022). In addition, methionine gama-lyase (MGL) has antibiotic properties, which can also be used to inhibit the growth of many warm bacteria and use positive and negative pathogenics. Due to the mechanism of action of methioninase in the antibiotic properties related to thiocyanates, this enzyme does not create antibiotic resistance (Raboni et al., 2024).

L-methioninase is found in nearly all organisms except mammals. Most bacteria, protozoans, archaea, and plants produce intracellular L-methioninase, while fungal sources produce extracellular L-methioninase (Irajie et al., 2021).

L-asparaginase and L-arginine are drugs widely used to treat lymphosarcoma, acute lymphoblastic leukemia, Hodgkin’s disease, and other types of tumors. However, one of the issues with its administration is that it can cause anaphylactic reactions (Kaiser, 2020). L-methioninase, on the other hand, does not have this problem. To produce L-methioninase, researchers have tried using fungi and bacteria as sources. While using fungi to produce L-methioninase is an attractive option, the risk of anaphylactic reactions caused by fungal sources is a concern. Selecting bacteria as producers of L-methioninase is an exciting prospect (Sahoo and Sahoo, 2021).

The design and optimization of biological processes are vital for improving proficiency and economic bioprocessing. Response Surface Methodology (RSM) is an exploited modeling procedure to establish the association between the responses and the independent variables. It can analyze the impacts of each variable itself or in combination via regression analysis. So far, Bax-Behken and RSM have been widely used to optimize the construction of many enzymes (Banihashemi et al., 2016).

This study mainly focuses on screening for L-methionine-producing bacteria from soil and then optimizing the culture media for enhanced enzyme production using statistical design. This is the first report of the three bacteria included *Pseudomonas mosselii*, *Ralstonia solanacearum, and Cytobacillus kochii* as a novel L-methioninase producer, which could be an optimistic alternative resource for cancer treatment.

## 2 Materials and methods

### 2.1 Sample collection

The soils were collected from various gardens in Iran, placed in sterile bags, and transported to the laboratory, where they were refrigerated at 4°C for further experimentation.

### 2.2 Screening for L-methionine producers

To begin with, various soil samples were cultured on a Nutrient Agar medium to isolate bacteria. Subsequently, a pure bacterial culture was suspended in 20 mL of modified M9 medium broth containing 0.1% methionine. This solution was then incubated at 30°C for 72 h at 120 rpm.

### 2.3 Enzyme assay

L-methioninas activity evaluated by Nessler’s method (Wriston and Yellin, 1973). 72-h bacterial cultures were centrifuged at 20124 RCF for 10 min, and a clear supernatant was kept for enzyme assay. 100 μL of supernatant transported to the sterile 1.5 mL microtube with 900 μL 0.5%W/V L-methionine (substrate) in Tris–HCl buffer (50 mM and pH 7.2). After incubation at 30°C for 60 min, 100 μL 15% trichloroacetic acid was added for stop the reaction and centrifuged at 8944 RCF for 10 min. The ammonia liberation in the supernatant was determined using the colorimetric technique by adding 200 Nessler regent in 200 μL supernatant and distilled water. The sample was first vortexed for 15 s and incubated at room temperature for 10 min, and finally, the absorption (OD) was measured at 425 nm. The ammonia released in the reaction was calculated based on the standard curve obtained using ammonium chloride. One unit of L-methioninase activity is defined as the amount of enzyme that releases 1 μL of ammonia per minute at 37°C.

### 2.4 Molecular identification of bacteria

The morphological, physiological, and biochemical characterizations of the best L-methionine-producing isolates (according to the highest yield of enzyme production at the screening stage) were determined by identification tests according to Bergey’s manual of systematic bacteriology (Sneath et al., 1986). For further identification, the analysis of the 16 s rDNA gene sequence using universal bacterial primers, 5′-CAGCCGCGGTAATAC-3′ as the forward primer and 5′-ACGGGCGGTGTGTAC-3′ as the reverse primer. Trench sequencing was employed to analyze 16S rDNA sequences After that, the phylogenic method for identifying bacterial isolates was used using the UPGMA algorithm tree in the Clustal W server.

### 2.5 Optimization of essential components

The optimization of medium constituents for L-methioninase production by the selected bacterium was performed in two steps. MINITAB software conducted all experimental designs and data analysis (version 20, PA, United States).

#### 2.5.1 Identification of essential nutrient components

Optimization of the conditions and compositions of the culture medium was carried out using the method of “one factor at a time,” in which many media components were tested to obtain the best results. In this research, the carbon and nitrogen sources (each at 0.2, 0.4, 0.6, and 0.8% w/v) as well as temperature (25°C, 30°C, 32°C, 37°C, and 40°C) and pH (Raboni et al., 2024; Irajie et al., 2021; Kaiser, 2020; Sahoo and Sahoo, 2021; Banihashemi et al., 2016; Wriston and Yellin, 1973; Box and Behnken, 1960; Selim et al., 2015) were assessed to screen and identify the critical medium components for L-methioninase production. It should be noted that all experiments were performed in the M9 medium, and the best results of the factors in each step were applied in the next step.

#### 2.5.2 Optimization of screened components

The next stage in the L-methioninase production optimization was to determine the most favorable level of each critical, independent variable as determined by the “one factor at a time” methodology. An experimental design, such as the Box–Behnken design (Box and Behnken, 1960), a fraction of the full factorial was used. Unlike the “one factor at a time,” this design assessed the quadratic effects and two-way interactions between the variables and determined the response’s nonlinear nature. The four independent variables were designed using MINITAB software (version 20, PA, United States). Experiments were performed in triplicate, and mean values were given. Finally, the final optimum experimental parameters were calculated using the Minitab Response Surface Optimizer function, which determined the best combination of each parameter.

## 3 Results and discussion

### 3.1 Isolation and screening of L-methioninase-producing bacteria

The bacteria were cultured in a modified M9 medium, and the screening of bacteria produces L-methionine. We cultured 16 samples from different soil sources in broth-modified M9 medium for 72 h. The experiments were repeated three times, and the average values were used. The results show that we found three isolates that can produce L-methionine, and the B5s9 isolate had higher activity than the other isolate. B5s9 isolate could be considered an excellent L-methioninase producer.

### 3.2 Identification of the isolated strains

**Figure 1:**
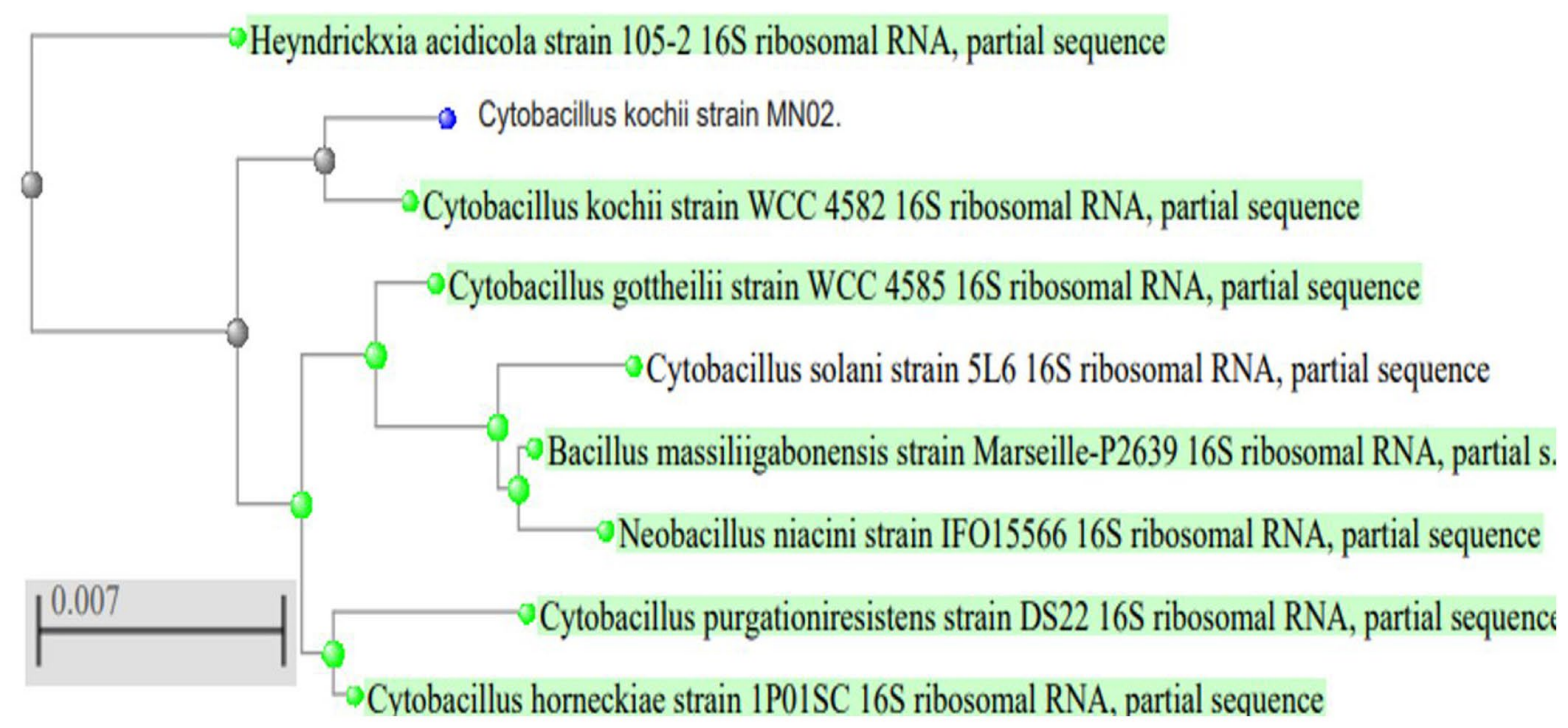
*Pseudomonas mosseli* strain MN02 16 S rRNA phylogenetic tree.

**Figure 2:**
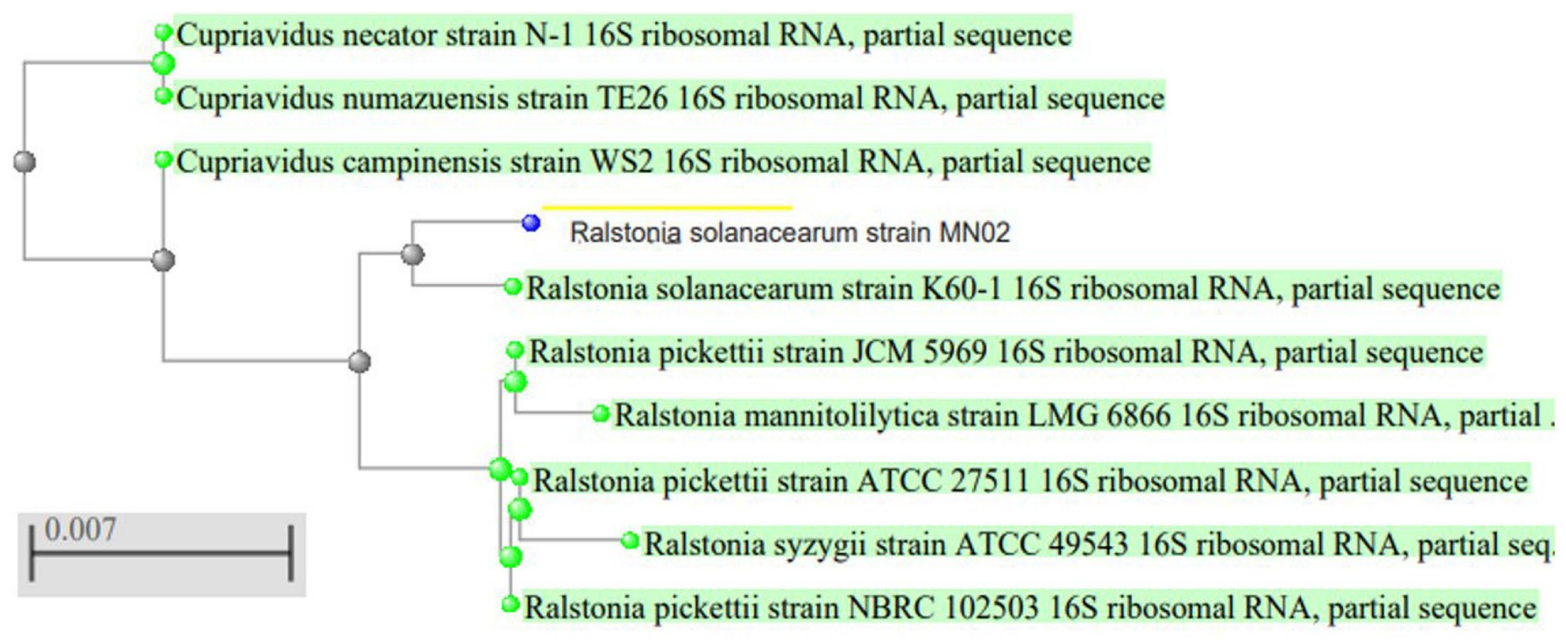
*Ralstonia solanacearum* strain MN02 16 S rRNA phylogenetic tree.

**Figure 3:**
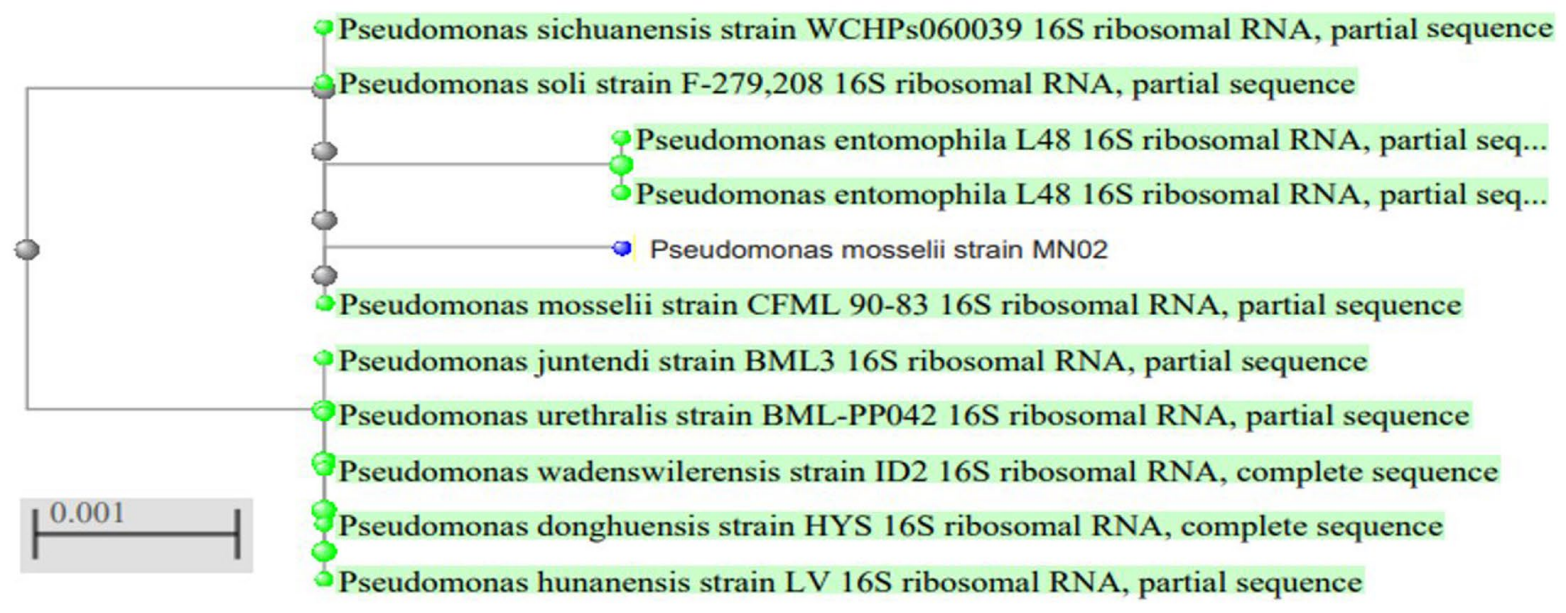
*Cytobacillus kochii* strain MN02 16 S rRNA phylogenetic tree.

To identify the isolated strain, the results demonstrated that the strain B5s8 was a gram-negative motile bacillus in addition to indole production; however, this isolate could not ferment all the tested carbohydrates. In parallel, the widely used method of 16 S rRNA gene analysis was also applied, demonstrating that the almost complete sequence of 16 S rRNA gene had 95% homology with *Pseudomonas mosselii strain MN02*. Moreover, the phylogenetic tree was drawn (Figure 1). Therefore, according to the observations and analyses, the strain MN02 was identified as *Pseudomonas mosselii.* Another strain that we placed as producing the L-methioninase includes B2s8 and NB7s3.B2S8 was a gram-negative andNB7s3 was gram-positive, and we used 16 S rRNA gene was applied, however after a complete sequence of 16 S rRNA gene, respectively, had homology with *Ralstonia solanacearum* strain MN02 and *Cytobacillus kochii* strain MN02 and drowned phylogenetic tree (Figures 2, 3).

### 3.3 Screening important media components for L-methioninase production by *Pseudomonas mosselii* sp. *MN02*

**Figure 4:**
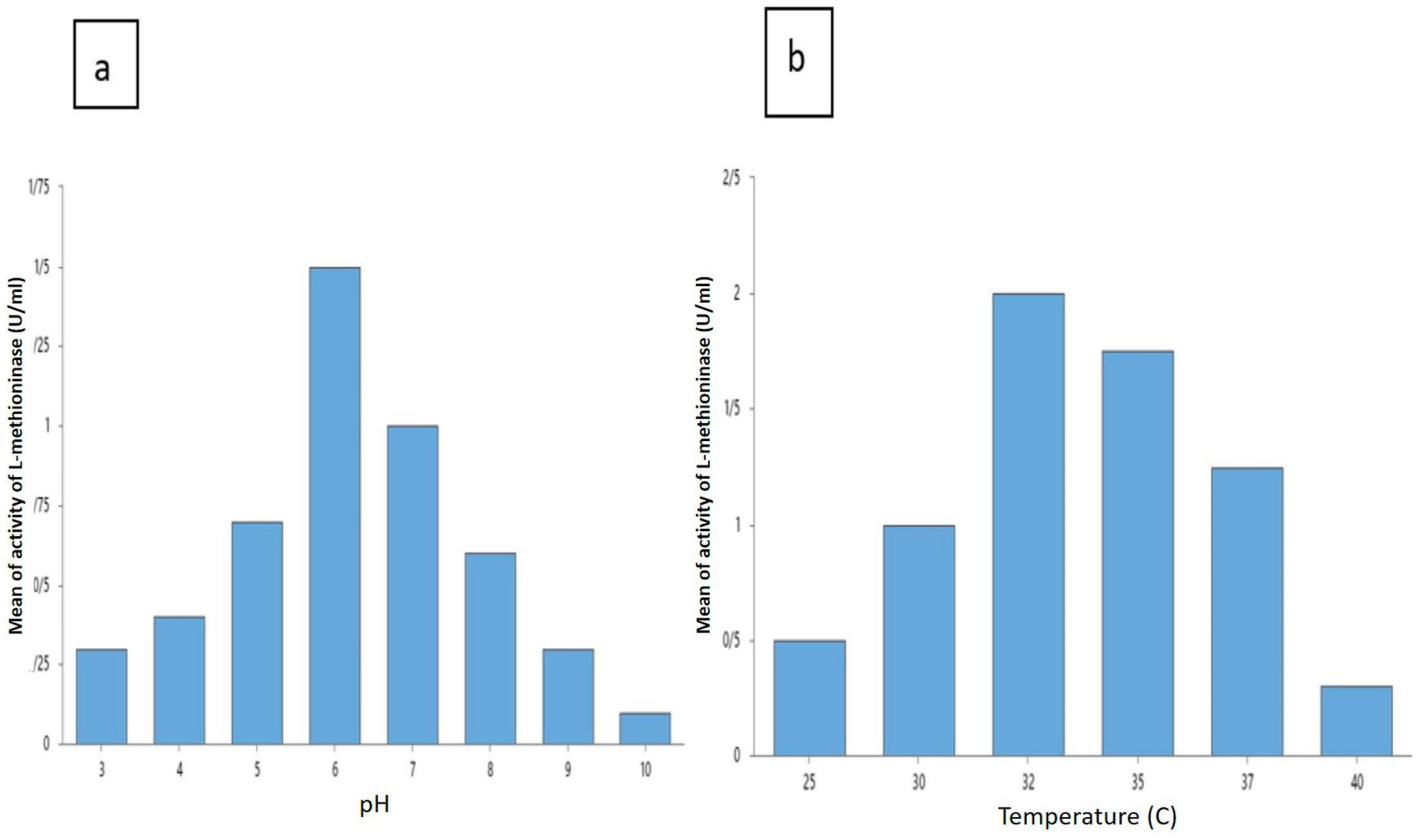
Effect of various factors pH **(A)** and temperature **(B)** on L-methioninase production.

L-methininase productions were assessed at various pH to discover the influence of pH. Maximum of L-methininase production of 1.5 ± 0.1 U/mL was obtained at a pH of 6. A decrease in the production of L-methioninase is observed in pH’s lower than 5 and higher than 8. Therefore, the optimum pH for *Pseudomonas mosselii* to produce L-methioninase has attained a pH of 6 (Figure 4A). In this regard, some scientists reported the best pH for L-methioninas production in other strains. Selim et al. reported that the best pH range for L-methioninase producing in *Streptomyces* sp. was 7–7.5 (Selim et al., 2015), and Mokham et al. reported that the best pH range for L-methioninase producing in *Alcaligenes* when increased pH from 4 to 6 (Mohkam et al., 2020). To study the influence of temperature for maximum production of L-methioninase was 32°C and L-methioninase production was 2.0 ± 0.1 U/mL. When we increased the temperature to more than 37°C, we could see an apparent decrease in enzyme production (Figure 4B).

**Figure 5:**
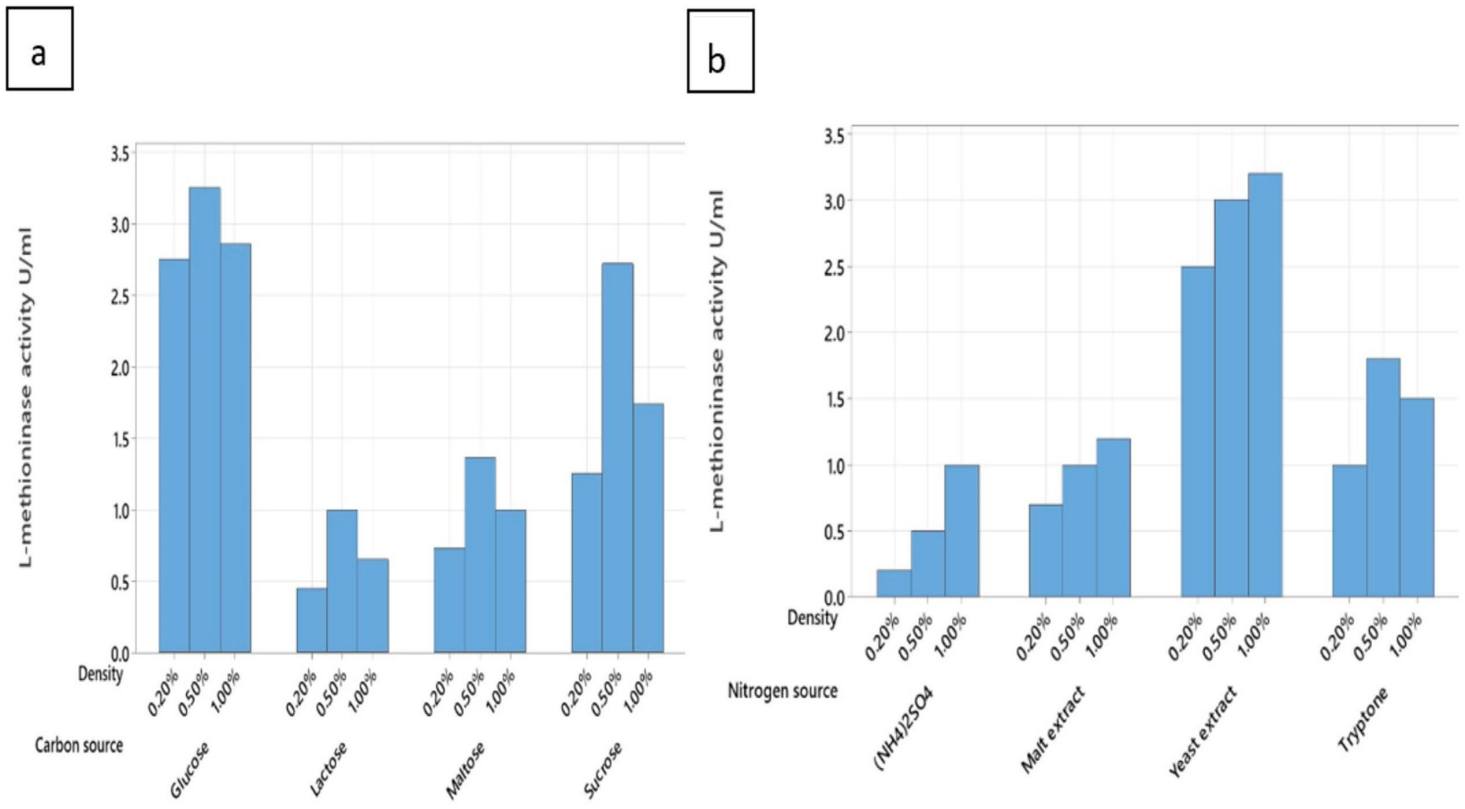
Effect of various factors carbon source **(A)** and nitrogen source **(B)** on L-methioninas production.

Among various nitrogen sources, we tested two different types of nitrogen sources, including inorganic and organic. The review and result of this are in Figure 5A. We found that the best nitrogen source for *Pseudomonas mosselii* to produce L-methioninase was yeast extract at 1% concentration, and MN02 can produce 3.2 ± 0.1 U/mL L-methioninas. After the yeast extract, the tryptone was placed at a concentration of 0.5%. On the other hand, we can see lower production of the enzyme in (NH4)2SO4; it can be shown that producing of L-methioninase in *Pseudomonas mosselii,* was not dependent on L-methionine which means that these bacteria can produce the enzyme in the absent of L-methionine (Singh and Kharayat, 2018). Some other bacteria identified in the past, like these bacteria, were not dependent on L-methionine for enzyme production, like *Alcaligenes* (Mohkam et al., 2020). However, fungi can produce L-methininase when L-methionine is in their culture. This fungi like *Trichoderma harzianum and Aspergillus* spp. (Salim et al., 2019). Without needing L-methioninase, this feature helps us produce L-methioninas from *Pseudomonas mosselii*, a cheaper nitrogen source. The properties of *P. mosselii*, including its faster growth and production compared to fungi, simpler cultivation conditions that are typically easier and more cost-effective, easier genetic engineering capabilities, and fewer side effects, produce L-methioninase without L-methionine, make it a more suitable choice for producing the enzyme L-methioninase.

The influence of different carbon sources, glucose was the best carbon source for L-methioninase production (3.25 ± 0.1 U/mL), followed by 0.5% concentration (Figure 5B). Like other studies that have been done before, it shows that in lack of carbon source, L-methioninase production was inhibited. This is significant for bacteria like *Alcaligenes* and *Acromobacter starkeyi.* Despite our findings, some bacteria produce more L-methioninanase with lactose source, which is probably related to the *β*-galactosidase gene in those bacteria like *Alcaligenes* (Mohkam et al., 2020).

### 3.4 Optimization condition for production of L-methioninase using response surface methodology

We tested four measure conditions such as pH, temperature, glucose, and yeast extract, and we tried to optimize these conditions to the optimum. We got help from Box–Behnken design and applied many conditions tabulated in Table 1. The variance analysis (ANOVA) result is tabulated in Table 2. The variable is considered significant when the *p* value is lower than 0.05. ANOVA showed the significance of all four factors (pH, temperature, glucose, and yeast extract) in producing L-methioninase. However, the interactive effects had no significant impact on L-methioninase production. We analyzed the data in multiple regression and found the model for predicting L-methioninase.


$$Y=13.07–0.4266X1-0.1160X2+2.071X3+2.218X4-0.760X3^{*}X4$$

**Table 1:** The results of an experimental study and the predicted values of L-methioninase production using the Box–Behnken design.

Run	Yeast extract (%)	Glucose (%)	temp	pH	Enzyme activity(U/ml)	Predicted value(U/ml)
1	1.5	1.5	30	6	11.0	11.7968
2	0.7	1.5	32	8	9.3	9.5908
3	2.0	1.5	40	10	8.7	9.1814
4	1.5	0.7	30	6	11.2	11.1604
5	0.7	0.7	32	8	8.1	8.9545
6	2.0	0.7	40	10	9.3	8.5450
7	1.5	2.0	30	6	13.4	12.2850
8	0.7	2.0	32	8	10.0	10.0791
9	2.0	2.0	40	10	9.5	9.6695
10	1.5	1.5	32	6	11.5	11.7710
11	1.5	1.5	40	6	9.9	10.6621
12	0.7	1.5	32	6	10.5	10.4069
13	0.7	1.5	30	8	9.8	9.61669
14	0.7	1.5	40	8	9.8	8.4819
15	2.0	1.5	30	6	12.8	12.0890
16	2.0	1.5	32	6	12.6	12.0632
17	1.5	0.7	32	8	10.3	10.3187
18	1.5	0.7	40	8	9.2	9.20974
19	1.5	0.7	32	10	9.7	9.36183
20	1.5	0.7	40	10	8.3	8.25289
21	0.7	0.7	30	6	10.0	9.79628
22	0.7	0.7	40	10	7.5	6.88873
23	0.7	0.7	30	10	7.3	8.02347
24	0.7	0.7	40	6	8.2	8.66154
25	2.0	0.7	30	6	11.4	11.4526
26	2.0	0.7	30	8	10.3	10.6366
27	2.0	0.7	32	6	11.8	11.4268
28	2.0	0.7	32	8	10.7	10.6108
29	1.5	2.0	32	8	12.6	11.4432
30	1.5	2.0	40	8	10.5	10.3343
31	1.5	2.0	40	10	9.3	9.37743
32	1.5	2.0	32	10	9.8	10.4864
33	1.5	1.5	30	8	10.9	10.9808
34	1.5	1.5	40	8	9.3	9.84610
35	1.5	1.5	40	10	8.5	8.88925
36	1.5	1.5	30	10	10.8	10.0240
37	2.0	2.0	30	6	12.0	12.5771
38	2.0	2.0	32	6	12.4	12.5513
39	2.0	2.0	32	8	11.5	11.7354
40	2.0	2.0	30	8	11.3	11.7612

**Table 2:** ANOVA test for L-methioninase activity from *Pseudomonas mosselii* strain MN02.

Source	DF	Adj SS	Adj MS	F-Value	P-Value
Regression	8	62.809	7.8512	37.25	<0.001
pH	2	16.496	8.2481	39.13	<0.001
temp	2	4.775	2.3874	11.33	<0.001
Yeast extract (%)	2	12.706	6.3531	30.14	<0.001
Glucose (%)	2	6.209	3.1045	14.73	<0.001
Error	31	6.535	0.2108		
Total	39	69.344			

X1, X2, X3, and X4 represent the coded values of pH, temperature, glucose (%), and yeast extract (%), respectively.

According to analysis, the significant *p* value (<0.001) and high *F* value showed that the model equation was in excellent agreement with the experimental. The coefficient of determination (*R*^2^) was 0.852 for L-methioninase production, which describes a higher correlation between experimental results and their predictions. The closer *R*^2^ is to 1.0, the more robust the model is and the better its prediction.

**Figure 6:**
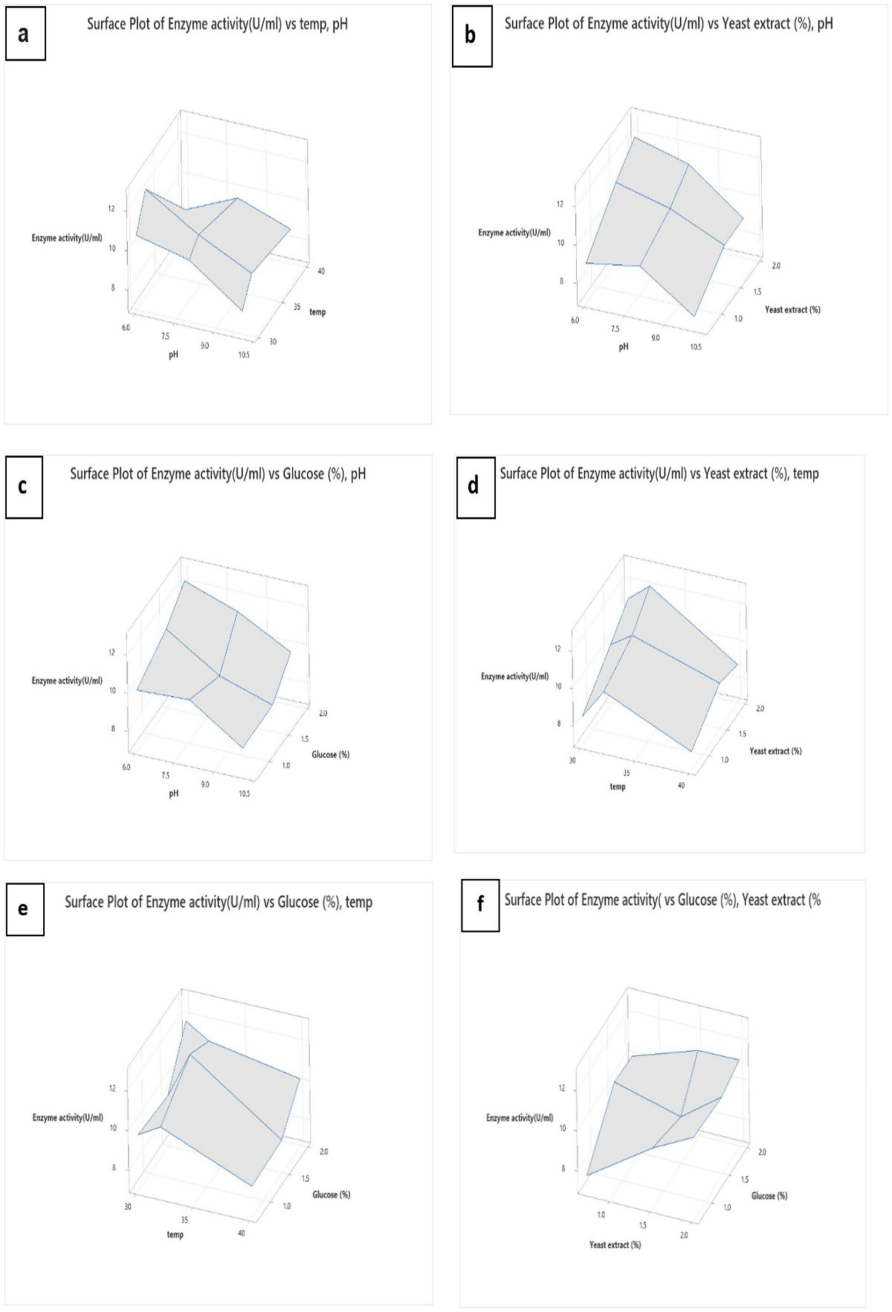
Surface plot of L-methioninase production, pH and temperature **(A)**, pH and yeast extract **(B)**, pH and glucose **(C)**, temperature and yeast extract **(D)**, temperature and glucose **(E)**, and yeast extract and glucose **(F)**.

After that, the surface plot was immersed in different conditions to determine the best conditions for L-methioninase production (Figures 6A–F). To identify the optimized values of the parameters, we created plots for the pairwise combination of parameters while keeping the other parameters constant at their middle level. Although the response surface plots helped evaluate the parameters, assessing them all simultaneously took work. Hence, we used Minitab’s Response Surface Optimizer function to determine the optimal levels of yeast extract (2.0%), glucose (2.0%), pH (Sahoo and Sahoo, 2021), and temperature (30°C). The model has predicted that L-methioninase will have an activity of 12.56 U/mL. The excellent correlation between the forecasted and examined values of the Box–Behnken design (as presented in Table 2) confirms the validity of the response model and the existence of an optimal point. In a similar study, Kharayat et al. used the RSM technique to optimize L-methioninase production in *Bacillus subtilis*, achieving 17.4 U/mL under optimized conditions. According to Sundar et al., the optimized conditions from *Serratia marcescens* resulted in an optimum activity of 13.7 U/mL, matching with Mokhom et al. from *Alcaligenes* (Mohkam et al., 2020).

We conducted several experiments to examine various factors affecting the production of L-methioninase. Due to the complexity of the process and the large number of experiments, we performed half of the experiments under different conditions. Our experiments found that the highest production of L-methioninase occurred when yeast extract was used at a concentration of 1.5%, glucose at 2.0% at a temperature of 30°C, and a pH level of 6. We used a Multiple Regression software called MiniTab to determine a model for predicting enzyme activity under different conidiation conditions. The model was highly accurate (86.63%) in our experiments. We then utilized this model to identify the optimal conidiation conditions for producing enzymes from *Pseudomonas mosselii*. According to the model, the best conidiation conditions are pH = 6, temperature = 30°C, glucose = 2%, and yeast extract = 2%, which produced 12.5670 U/mL of L-methioninase.

## 4 Conclusion

The current study has presented new bacterial strains for producing L-methioninase. Three bacterial genera, namely *Pseudomonas mosselii* spp. MN02, *Ralstonia solanacearum* spp. MN02, and *Cytobacillus kochii* spp. MN02, were isolated and identified from soil samples. We used a combination of one-factor-at-a-time (OFAT) and Box–Behnken experimental approaches to optimize the production of L-methioninase from the best producer among these three bacterial species. We analyzed the components of the environment and modeled the data, resulting in a significant increase in L-methioninase production. The Box–Behnken approached proved particularly effective in developing a mathematical model to predict the optimal placement of materials. The high *R*^2^ value of 0.852 for L-methioninase activity indicated the effectiveness of this method, which was further validated by a model accuracy of 86.63%. Initially, the production of L-methioninase by *Pseudomonas mosselii* spp. MN02 from the primary culture medium was measured at 3.25 ± 0.1 U/mL. After optimizing the culture conditions—pH at 6, temperature at 30°C, glucose at 2%, and yeast extract at 2%—the production increased to 12.5670 U/mL. This substantial increase demonstrates that *Pseudomonas mosselii* spp. MN02 could be a promising source of L-methioninase for potential use in anticancer therapy. This study highlights a new strategy for optimizing L-methioninase production, which could have significant implications for cancer treatment research.

## Data Availability

The original contributions presented in the study are included in the article/supplementary material. Further inquiries can be directed to the corresponding author.
